# Compositional and genetic alterations in Graves’ disease gut microbiome reveal specific diagnostic biomarkers

**DOI:** 10.1038/s41396-021-01016-7

**Published:** 2021-06-02

**Authors:** Qiyun Zhu, Qiangchuan Hou, Shi Huang, Qianying Ou, Dongxue Huo, Yoshiki Vázquez-Baeza, Chaoping Cen, Victor Cantu, Mehrbod Estaki, Haibo Chang, Pedro Belda-Ferre, Ho-Cheol Kim, Kaining Chen, Rob Knight, Jiachao Zhang

**Affiliations:** 1 Department of Pediatrics, University of California San Diego, La Jolla, CA USA; 2 Center for Microbiome Innovation, Jacobs School of Engineering, University of California San Diego, La Jolla, CA USA; 3 School of Food Science and Engineering, Hainan University, Haikou, China; 4 Department of Endocrinology, Hainan General Hospital, Hainan Affiliated Hospital of Hainan Medical University, Haikou, China; 5 Hubei Provincial Engineering and Technology Research Center for Food Ingredients, Hubei University of Arts and Science, Xiangyang, Hubei province China; 6 Key Laboratory of Food Nutrition and Functional Food of Hainan Province, Haikou, China; 7 Department of Bioengineering, University of California San Diego, La Jolla, CA USA; 8 Scalable Knowledge Intelligence, IBM Research-Almaden, San Jose, CA USA; 9 Department of Computer Science and Engineering, University of California San Diego, La Jolla, CA USA

**Keywords:** Microbiome, Biomarkers, Population genetics

## Abstract

Graves’ Disease is the most common organ-specific autoimmune disease and has been linked in small pilot studies to taxonomic markers within the gut microbiome. Important limitations of this work include small sample sizes and low-resolution taxonomic markers. Accordingly, we studied 162 gut microbiomes of mild and severe Graves’ disease (GD) patients and healthy controls. Taxonomic and functional analyses based on metagenome-assembled genomes (MAGs) and MAG-annotated genes, together with predicted metabolic functions and metabolite profiles, revealed a well-defined network of MAGs, genes and clinical indexes separating healthy from GD subjects. A supervised classification model identified a combination of biomarkers including microbial species, MAGs, genes and SNPs, with predictive power superior to models from any single biomarker type (AUC = 0.98). Global, cross-disease multi-cohort analysis of gut microbiomes revealed high specificity of these GD biomarkers, notably discriminating against Parkinson’s Disease, and suggesting that non-invasive stool-based diagnostics will be useful for these diseases.

## Introduction

Graves’ disease (GD) is an autoimmune disorder that frequently results in hyperthyroidism. In regions with sufficient iodine intake, GD’s prevalence is about 0.5%, with annual incidence of 20–50 cases per 100,000 people [1]. GD prevalence is sex-specific: 3% in females but 0.5% in males during their lifespan [2]. Weight loss, fatigue, anxiety, heat intolerance, tremor, and palpitations are the most common symptoms, occurring in >50% of patients [3]. Unambiguous identification of the factors underlying GD has not yet been accomplished, increasing the difficulty of disease treatment [1]. The diagnosis of hyperthyroidism is based on characteristic clinical features, serum thyrotropin, free thyroxine levels and thyrotropin receptor antibodies. Although current diagnostics are sufficient for the most severe patients, the clinical feature and blood index-based diagnosis is complex and time-consuming, and usually delays early diagnosis and treatment for mild GD patients. Both GD patients and Parkinson’s patients have common clinical and biochemical diagnostic features that make differential diagnosis harder and mask the appearance of one of these disorders during the course of the other [4]. Accordingly, a more convenient and accurate diagnosis method for GD is urgently needed.

Recent studies have highlighted the essential role of the gut microbiome in maintaining the immune system and human health. Autoimmune disorders are a category of diseases in which normal cells, tissues and organs are mistakenly targeted by the immune system. An increasing amount of evidence revealed the close relationship between intestinal microbes and various metabolic and autoimmune diseases, including type 2 diabetes [5], liver cirrhosis [6], polycystic ovary syndrome [7], gout [8] and even mental or nervous system disorders such as Alzheimer’s diseases [9] and Parkinson’s disease (PD) [10]. Accordingly, disorders of the gut microbiome, as well as numerous chronic disease-specific microbial biomarkers, are being identified, and treatment strategies targeting gut microbes are considered a promising new approach to diagnosis and treatment.

GD-represented autoimmune thyroid disease is the most prevalent organ-specific autoimmune disease. Altered microbiota composition in the gut, as well as the decreased microbial products particularly short-chain fatty acids (SCFAs), promotes the development of autoimmune thyroid disease by several hypothesized mechanisms including controlling the integrity of intercellular junctions and the microbial transcriptomic, proteomic, and metabolic changes [11]. Several studies have focused on the microbial taxonomic disorder of GD patients, and revealed a higher abundance of intestinal *Prevotellaceae* and *Pasteurellaceae* in GD patients, whereas *Enterobacteriaceae*, *Veillonellaceae*, and *Rikenellaceae* decreased significantly in GD [12, 13]. However, the small sample size and limited resolution of the 16S rRNA amplicon sequencing technology limited the universality and significance of these studies. Larger cohorts and integration of different levels of analysis from high-resolution shotgun metagenomics data were therefore needed to explore the GD patients’ alteration in gut microbial taxonomy, genes, pathways and functions, metabolites, and mutational spectra, to establish robust microbial biomarkers at these levels for GD diagnosis.

**Fig. 1:**
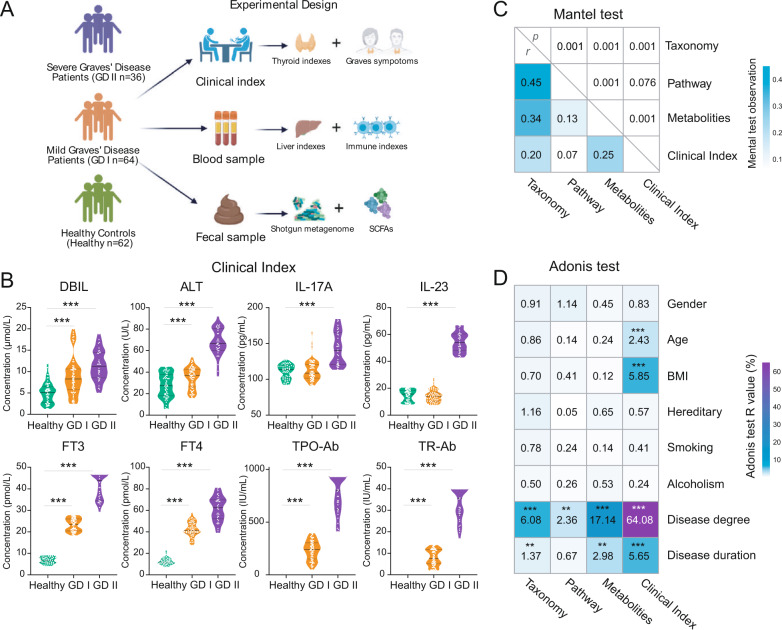
Experimental design and integrated analysis of the Graves’ disease microbiome. **A** The experimental design. A total of 162 human subjects were divided into three groups according to disease states: healthy control (Healthy), mild Graves’ disease (GD I) and severe Graves’ disease (GD II). Shotgun metagenomic sequencing was applied to perform microbiome analyses of fecal samples, while multiple clinical indexes were examined. **B** The violin plots showing the differential distributions of clinical indexes among three host groups. **C** The Mantel tests quantifying the correlation between each pair of measurements (taxonomic profile, functional profile, predicted metabolite profile and clinical indexes) from host individuals. The values in the lower triangle indicate the Mantel *R* statistics, which range from −1 to 1, representing the correlation between a pair of measurements. The corresponding *p* values of the correlations are shown in the upper triangle. **D** The Adonis test showed that Graves’ Disease is the dominant factor contributing to the variation in the intestinal microbiome of human subjects. Asterisks: statistical significance (*p ≤ 0.05, **p ≤ 0.01, ***p ≤ 0.001).

To address these challenges, we recruited 162 subjects and divided them into three groups: healthy controls (Health), mild Graves’ patients (GD I) and severe Graves’ patients (GD II) according to their clinical indexes (Fig. 1A). Shotgun metagenomic sequencing and inferred metabolomics were applied to describe the intestinal microbial characteristics and microbial mutations of GD patients. Then, combined biomarkers were identified from machine learning, including specific metagenomic species, metagenome-assembled genomes (MAGs), MAG-derived genes and single-nucleotide polymorphisms (SNPs). Finally, we performed a multi-cohort analysis to confirm the specificity of these biomarkers across different metabolic and autoimmune diseases. This work extended our understanding of the microbial ecology of GD pathogenesis, and developed a useful predictive model for GD diagnosis based on intestinal microbial biomarkers.

## Results

### Graves’ Disease (GD) alters the gut microbiota and its functions

In the present study, 162 subjects were divided into three groups: the healthy control group (Healthy, *n* = 62), mild Graves’ patient group (GD I, *n* = 36) and severe Graves’ patient group (GD II, *n* = 64) (Fig. 1A). Eleven clinical indexes characterizing the conditions of the thyroid, liver, and immune system were determined for each subject. Significant differences (*p* ≤ 0.001, Wilcoxon rank-sum test, two-tailed) were found in all clinical indexes between Healthy and GD II, and in liver and thyroid indexes between Healthy and GD I (Fig. 1B). Shotgun metagenomic sequencing was performed on feces from each subject to assess the gut microbiome composition. Metagenomes were assembled and functional genes were annotated, and the putative metabolic capacities of the microbiomes were estimated by MelonnPan (model-based genomically informed high-dimensional predictor of microbial community metabolic profiles) pipeline.

We performed a combined analysis of metagenome-derived taxonomic and functional profiles, as well as clinical indexes. Mantel tests across these data types indicated tight coupling between the intestinal bacterial profile and the metabolic pathways, metabolites and clinical indexes (Fig. 1C). Adonis test of major metadata variables vs individual data matrices confirmed the dominant impact of the disease status (Healthy, GD I and GD II) on the clinical indexes (*R*^2^ = 64.08). Meanwhile, it also revealed strong associations between GD status and intestinal bacteria (*R*^2^ = 6.08), and their metabolic potentials (*R*^2^ = 17.14). In comparison, other demographic, behavioral and clinical variables have no significant association with any metagenome-derived metrics (Fig. 1D).

### Alteration of intestinal microbiome and predicted metabolites in GD patients

**Fig. 2:**
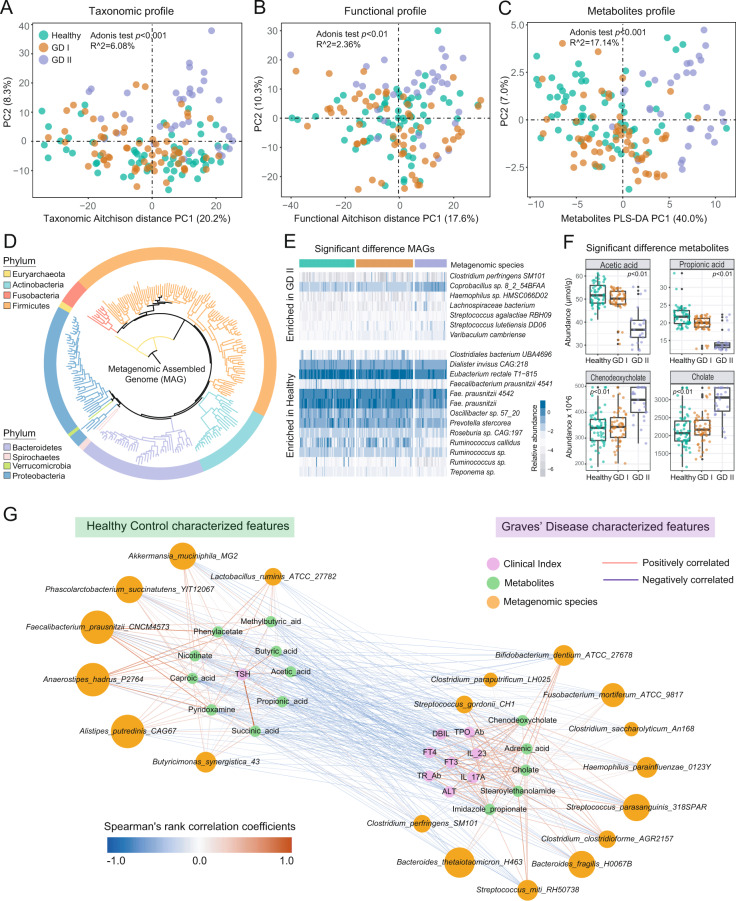
The Alteration of intestinal microbiome and microbial metabolites in GD patients. Principal coordinates analysis (PCoA) based on Aitchison distances of microbial species (**A**), and functional features (**B**). Each point in the PCoA plots represents a host subject in healthy, mild (GD I) or severe (GD II) Graves’ disease groups. The colors of points represent the host groups. **C** Partial least squares-discriminant analysis (PLS-DA) based on the microbial metabolites predicted from the metagenomic data. **D** Phylogenetic tree of the MAGs with clades colored by phylum. **E** MAGs of significant difference between the healthy and the GD groups. **F** The intestinal microbial metabolites that differed significantly between healthy and GD II groups (Wilcoxon rank-sum tests, two-tailed). **G** The network analysis of MAG markers, predicted metabolite and clinical indexes. The microbe-metabolite interactions were quantified by their Spearman’s rank correlation coefficients to exhibit the correlation between the intestinal microbiome and Graves’ disease. The edge widths and colors (red: positive correlated and blue: negative correlated) are proportional to the correlation strength. The node sizes are proportional to the mean abundance in the respective population.

We constructed PCoA ordinations based on Aitchison distance (Fig. 2A) and Bray–Curtis dissimilarity (Fig. S1A) among the taxonomic profiles. Surprisingly, intestinal microbiota of subjects in the Healthy and GD I groups were similar but obviously separated from the patients in the GD II group. To quantify these differences, we performed the Adonis test and calculated the *R*^2^ (*R*^*2*^ = 6.08) and *p* values (*p* < 0.001), which indicated a serious disorder in the intestinal microbiota of severe GD patients. The results were confirmed in the microbial alpha diversity aspect, in which we observed a sharp decrease in microbial alpha diversity of GD II patients (Fig. S1B).

Accordingly, we identified species with significant differences between the healthy and the GD II groups (Wilcoxon rank-sum test, two-tailed) in trend changes as the potential biomarkers (Fig. S1C). Specifically, *Faecalibacterium prausnitzii*, *Butyricimonas faecalis*, *Bifidobacterium adolescentis* and *Akkermansia muciniphila* decreased in the GD II group, whereas *Eggerthella lenta*, *Streptococcus parasanguinis*, *Veillonella parvula*, *Fusobacterium mortiferum* and *Streptococcus salivarius* were enriched.

We further assembled the metagenomic reads into contigs and constructed metagenomic assembled genomes (MAGs) in each subject. Phylogenomic analysis of the MAGs suggested overall consistency with taxonomic annotation (Fig. 2D and Fig. S2A). Then, we identified MAGs with significantly differential abundance among the three groups and constructed a heatmap with represented MAGs (Fig. 2E and Fig. S3A). Furthermore, we reassembled the significantly different MAGs for specific different genes identification and annotation (Fig. S3B).

After demonstrating the disorder in the intestinal microbiota of patients in GD II group, we further explored the changes in microbial metabolic pathways. Annotated by the UniRef protein database, we obtained profiles of microbial gene families and metabolic pathways. The Aitchison distances based on the functional features suggested an obvious shift in the intestinal microbial functional capacity of GD patients (PC1 between the Healthy and GD II groups, *p* < 0.05, Fig. 2B). Among the differentially abundant metabolic pathways between Healthy and GD II groups, mevalonate and isoprene biosynthesis, formaldehyde assimilation and allantoin degradation significantly increased in relative abundance in the severe GD patients, whereas the microbial metabolic abilities of fatty acid biosynthesis, creatinine degradation, pyruvate fermentation to hexanol, anaerobic energy metabolism and gluconeogenesis decreased significantly in relative abundance in the patients (Fig. S2B).

By performing the MelonnPan pipeline [14] based on the gene family profile inferred by the HUMAnN (v2.0) [15] pipeline (UniRef 90 database annotation), we predicted metabolomics profiles including >80 metabolites, including the SCFAs determined by GC-MS. Similarly, PLS-DA of metabolic profiles revealed obvious differences between GD II patients and healthy subjects (Fig. 2C). The metabolites with significant differential abundance, including acetic acid, propionic acid, cholate and chenodeoxycholate among the three groups were identified as potential biomarkers (Fig. 2F and Fig. S4).

Then, we constructed a network to visualize the correlation among the GD-associated MAGs, metabolites and clinical indexes based on Spearman’s rank correlation coefficients (Fig. 2G). The network reveals well-defined clusters separating healthy and GD-associated features. It characterizes the increase of pathogenic bacteria and opportunistic pathogens with detrimental metabolites as well as the lack of mutual microbes and organic acids were the common intestinal microbial compositional characteristic of GD patients.

### Differential genetic variations of GD-associated microbes

**Fig. 3:**
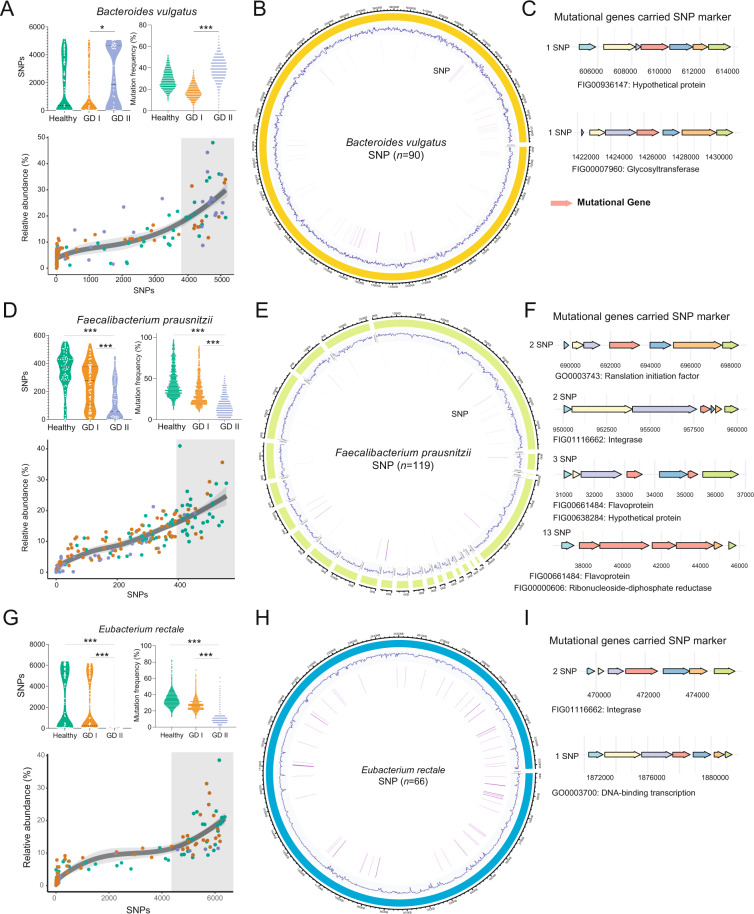
The SNP profile of the target species in each group. **A**, **D**, **G** (top panel) The number of SNPs annotated in the three species among the three groups, and the mutational frequency of each SNPs annotated in the three intestinal species. **A, D, G** (bottom panel) The correlation analysis revealed a high consistency between the relative abundance and the number of SNPs of the mutational species. **B, E, H** Genomic locations and contexts of SNPs in the species of *Bacteroides vulgatus* (**B**, *n* = 90), *Faecalibacterium prausnitzii* (**E**, *n* = 119) and *Eubacterium rectale* (**H**, *n* = 66), which exhibited the significant difference in mutational frequency between the healthy and GD groups. **C, F, I** The functions of the mutated genes (red) carried the SNP markers were annotated at the bottom panel.

Beyond the taxonomic and functional features, we further explored the evolutionary changes at the genetic level in intestinal microbial species. We aligned the metagenomic data against the reference genomes of species with relative abundance higher than 0.5% in the present cohort and reconstructed a profile of SNPs. A total of nine common intestinal species were annotated, with the number of SNPs ranging from 46 to 7603 (Fig. 3A, D, G and Fig. S5). Among them, 776 SNPs were annotated in the species of *Faecalibacterium prausnitzii*, 5974 in *Bacteroides vulgatus* and 7603 in *Eubacterium rectale*.

A larger number of SNPs indicative of higher evolutionary diversity was observed in the genome of *B. vulgatus* in GD patients, whereas the opposite was found in *F. prausnitzii* and *E. rectale* genomes (Fig. 3B, E and H). Further analysis revealed the consistency between the relative abundance and the number of SNPs of these species, (Fig. 3A, D and G).

Then, we compared the frequency of each SNP among the three groups, and calculated the *p* values of each SNP for the healthy group vs GD I group, healthy group vs GD II group and GD I vs GD II groups (Wilcoxon rank-sum test, two-tailed). The significantly different SNPs between the control and the GD groups were identified when: i, the *p* values of healthy group vs GD II group and GD I vs GD II groups were <0.05; ii, the mean frequency was in the order of: healthy group > GD I group > GD II group, or healthy group < GD I group < GD II group. Accordingly, 275 SNPs were identified as significantly different SNPs between the healthy and GD groups. They were annotated in the genome of *B. vulgatus* (*n* = 90), *F. prausnitzii* (*n* = 119) and *E. rectale* (*n* = 66). These SNPs are mainly located in genes encoding for of xylanase activity, mannonate dehydratase activity, beta-lactamase activity, transporter activity and beta-galactosidase activity (Fig. 3C, F, I).

### Combined microbial marker types predictive of GD status

**Fig. 4:**
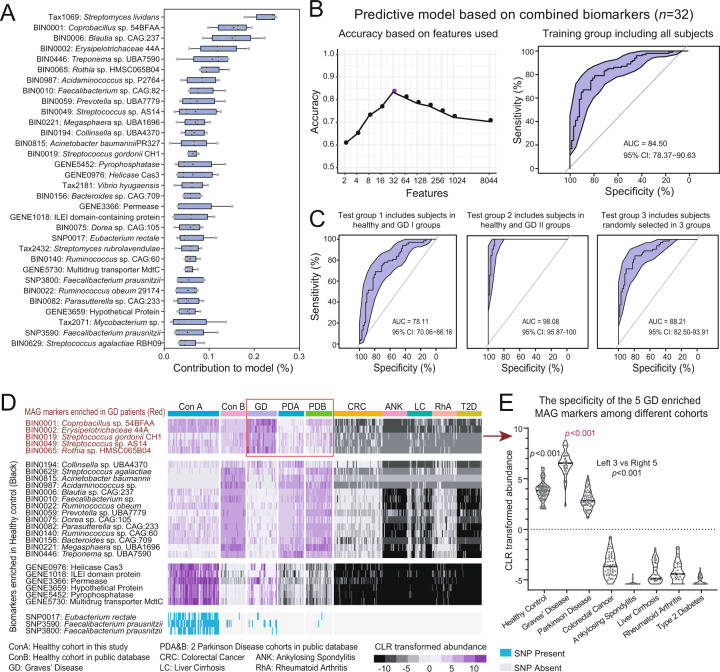
Identification of GD-associated biomarkers using a machine learning approach and the multi-cohort analysis reveals gut microbiome biomarkers that are specific to GD. **A** The importance of the biomarkers was ranked according to their contribution to the predictive model built by Random Forest. **B, C** The receiver operating characteristic (ROC) curve and the area under curve (AUC) both in the training and three test groups was calculated. **D** The heatmap shows the specificity of GD-associated MAG biomarkers to those commonly studied metabolic diseases: ankylosing spondylitis (ANK), liver cirrhosis (LC), colorectal cancer (CRC), Parkinson’s disease (PDA: a European cohort, PDB: a Chinese cohort), rheumatoid arthritis (RhA) and type 2 diabetes (T2D), as well as 2 healthy control cohorts (ConA and Con B). **E** The violin plot shows the quantitative difference in the total CLR-transformed abundance of the five marker MAGs across all the cohorts.

Using the Random Forest method, we trained a supervised classification model for the disease status based on the combination of intestinal species, MAGs, MAG-related genes and SNPs (Fig. 4A). The resulting predictive model with the highest accuracy (area under the receiver operating characteristic curve, or AUC = 84.50%) encompassed 32 biomarkers, including four species, 19 MAGs, six related genes and three SNPs (Fig. 4B). We ranked the 32 biomarkers according to their contribution to the predictive model. The predictive model was applied to three test sets and exhibited high accuracy (Fig. 4C). It effectively distinguished severe (AUC = 98.08%) and mild (AUC = 78.11%) Graves’ patients from healthy subjects, and determined the disease status from all three subject groups (AUC = 88.21%).

Meanwhile, we constructed three separate predictive models, each of which was based on one type of feature (MAGs, genes or SNPs). We found that 16 MAGs or 16 genes were needed to accurately classify the samples in the training set (AUC = 74.60% and 79.85%, respectively) (Fig. S6A, B), but as many as 64 intestinal microbial SNPs or 64 selected species could reach similar accuracy (AUC = 79.53%) (Fig. S6C). The results could be confirmed by the confusion matrix of the test group (Fig. S6D). However, the performance of the combined biomarkers from multiple feature types (see above) outperformed that of the biomarkers of any single type (AUC diff. 4.65–9.90%).

### Specificity of GD biomarkers against other metabolic diseases

To test the performance of the 32 combined GD biomarkers developed above in a broader background, we performed a multi-cohort analysis across another healthy cohort and six metabolic diseases cohorts including ankylosing spondylitis, liver cirrhosis, colorectal cancer, PD, rheumatoid arthritis and type 2 diabetes. The specificity of the combined biomarkers was calculated. Among the 32 biomarkers, we highlight the importance of five MAG markers under the family *Erysipelotrichaceae* and the genera *Coprobacillus*, *Streptococcus* and *Rothia* that are enriched in all GD patients (Fig. 4D, red), which exhibited unique specificity among all cohorts. The discrimination was quantified and confirmed by the violin plot constructed with the CLR-transformed abundance of the five MAGs across all cohorts (Fig. 4E).

Most notably, we observed that the five GD-enriched MAG markers also exhibited excellent discrimination in PD (Fig. 4D, **upper panel in red box**), a disease often involving thyroid dysfunction and is difficult to distinguish from GD in diagnosis [14]. To confirm this observation, we further validated the specificity of the five markers in two separate PD cohorts. The abundance of the five MAG markers was significantly higher in GD patients compared with that in PD patients (Fig. 4E). The predictive model constructed based on these five markers also exhibited high accuracy (AUC = 97.31%) in discriminating GD and PD subjects (Fig. S7).

## Discussion

In this study we explored the relationship between GD and gut microbiome. A combined analysis of shotgun metagenome and predicted metabolite data of fecal samples and clinical parameters of the subjects revealed a clear dysbiosis of the gut microbiome in severe GD patients, as compared with healthy controls and subjects with mild GD. The study provided evidence that such alteration is not shared with other diseases. We identified microbial species and metabolic pathways differentiating healthy subjects from patients, revealing a clear bipartite pattern in a co-occurrence network spanning the two subject groups. Specifically, some commensal microbes including *Faecalibacterium prausnitzii* and *Bifidobacterium adolescentis* [16, 17] and beneficial microbial metabolites such as SCFAs significantly decreased in the gut of severe GD patients. *F. prausnitzii* reportedly has anti-inflammatory properties and contributes to gut health through butyrate production [18]. Gut microbes such as *F. prausnitzii* and *Bifidobacterium* sp. are often referred to as “beneficial bacteria” because they exhibit health-promoting properties [16, 17]. It is proposed that elevated abundance of *F. prausnitzii* and *Bifidobacterium* sp. in the gut leads to increased production of SCFAs, which improve gut health by increasing the intestinal barrier function and reducing the translocation of bacterial endotoxins across the gastrointestinal wall, where they could cause inflammation and insulin resistance [5]. The significant depletion of *F. prausnitzii* and *Bifidobacterium* sp. is a typical feature of intestinal microbiota disorder, our observation of reduced *F. prausnitzii* and *Bifidobacterium* sp. and decline in SCFA is in line with these reports.

Analysis of different data layers derived from shotgun metagenomic data has provided an invaluable wealth of information for understanding the connections between human-associated microbiome and health conditions. To date, most of these efforts have focused on taxonomic units, functional modules, and predicted metabolic products [6, 19–21]. The evolutionary dynamics of microbial associates is largely overlooked. Despite that genetic variations of microbial genomes, such as SNPs and structural variants have long been noted [22], it was not until recently that researchers started to associate microbial genetic variations with host health [23, 24]. Here we explored the evolutionary changes at the molecular level in intestinal microbial species, and combined information with the classical intestinal microbial factors listed above. A large number of SNPs indicative of higher evolutionary diversity were observed in the genome of *B. vulgatus* in GD patients, and the opposite pattern in the genomes of *F. prausnitzii* and *E. rectale*. These mutations may be the driving force of the species colonization in the host gut, implicating the correlation between evolutionary elasticity and bacteria fitness [25], and further implicating the connection with the development of the disease, which is worth further validation using experimental approaches.

Biomarker discovery is the key goal in many microbiome studies as they implicate potentials for developing rapid, non-invasive diagnostic approaches. The complex nature of microbiome-host interaction dictates that single biomarkers revealed by classical correlation analyses are usually not adequate in predicting the phenotype. As of today, utilizing a comprehensive collection of microbiome features for machine learning analysis is popular and has shown its power in relating microbiome with health conditions [26, 27]. It has even been suggested that microbiome-based models outperform host genome-based models [28]. However, to our knowledge, such studies are so far limited to the use of a single marker type, such as taxonomic or functional units. However, to our knowledge, such studies are so far limited to the use of a single marker type, such as taxonomic or functional units. Only a few studies have attempted to combine biomarkers types. In one recent example, combined metagenomic and metabolomic markers were successful in discriminating major depressive disorder from healthy individuals. [29] Similarly, in this study, we combined four data layers derived from shotgun metagenomics: reference-based species assignments, reference-free MAGs, MAG-annotated genes, and SNPs, to construct a model that predicts GD status. Our model optimization analysis demonstrated that a combination of 32 biomarkers from all four feature types yielded the highest accuracy in both the training set and three different test sets, and this model notably outperformed models constructed and optimized using any single biomarker type. This interesting finding underscores the importance of integrating multiple layers of information for developing more accurate diagnostic models in microbiome studies.

Moreover, because a large number of microbial markers have been identified and reported in various diseases, the inevitable overlap could potentially challenge the specificity of prediction using those biomarkers, limiting their application in clinical use [30]. Accordingly, we further tested the performance of the GD biomarkers in a broader background, including seven cohorts of six other metabolic diseases and another healthy cohort. The GD biomarkers exhibited preferable specificity. In particular, our predictive model has high performance in distinguishing GD and PD. The two diseases share highly similar symptoms such as rigidity, hypokinesia, facial hypomimia, and voice abnormalities, causing diagnostic confusion in clinical practices, especially for elders suffering GD and/or PD [4, 31, 32]. Five GD-enriched MAGs related to genera *Coprobacillus* and *Streptococcus* exhibited excellent specificity. Detection of these biomarkers in fecal samples could potentially be a rapid and convenient diagnostic approach to determine whether a patient is suffering from GD or PD, in spite of the clinical observations shared by the two diseases.

## Conclusions

In this study we explored the relationship between GD and gut microbiome. A combined analysis of shotgun metagenome and predicted metabolite data of fecal samples and clinical parameters of the subjects revealed a clear dysbiosis of the gut microbiome in GD, patients, and provided evidence that such alteration is not shared with other diseases. A supervised classification model identified a combination of biomarkers including microbial species, MAGs, genes and SNPs, with predictive power superior to models from any single biomarker type (AUC = 0.98). This work extended our understanding of the microbial compositional and genetic of GD pathogenesis, and developed a useful predictive model for GD diagnosis based on intestinal microbial biomarkers.

## Materials and methods

### Experimental design and subject recruitment

All subjects were recruited from the Hainan Provincial People’s Hospital, Haikou, China. The subjects’ basic information (gender, age, BMI, smoking, and alcoholism) and clinical indexes were recorded in Table S1. Fecal samples were collected from each subject in the healthy group in the morning before the first meal. The cohort consisted of three groups: the healthy control group (Healthy, *n* = 62), the mild GD patient group (GD I, *n* = 64) and the severe GD patient group (GD II, *n* = 36) according to their thyroid-related diagnostic results (Fig. 1A). For each GD patient, their fecal and blood samples were collected by a doctor during their clinical visit. After the weight of the fecal materials was determined, a sample protector (CW0592M, CWBIO, China) was added at a ratio of five-to-one to the sample to stabilize nucleotides. The samples were stored at −20 °C until further processing.

### Ethics approval and consent to participate

The study was reviewed and approved by the Ethics Committee of the Hainan General Hospital (2018-109), and informed consent was obtained from all volunteers in written form before they were enrolled in the study. Sampling and all described subsequent steps were conducted in accordance with the approved guidelines.

### Clinical indexes determination

A total of 11 clinical indexes including alanine aminotransferase (ALT), direct bilirubin, free triiodothyronine (FT3), free thyroxine (FT4), thyroid-stimulating hormone (TSH), thyroid peroxidase antibodies, thyroid-stimulating hormone receptor antibodies and immune indexes interleukin-17A (IL-17A) and IL-23 were determined by using the enzyme-linked immunosorbent assay method.

### Short-chain fatty acid determination

As mentioned before, the SCFAs in the gut, which included acetic acid, propionic acid, butyric acid and valeric acid, were analyzed by the gas chromatography-mass spectrometry (GC-MS). Firstly, fecal samples were thawed, weighted, and diluted in isooctane for 1:10 (w/v). Secondly, fecal mixture was homogenized about 15 min into the suspension and centrifuged for 10 min with 5000 *g*. 500 μL supernatants were absorbed and dried with SpeedVac (Thermo Science) overnight. Then the dried extraction was dissolved in 50 μL methoxyamine hydrochloride solution. After adding 20 mg/mL pyridine, the mixture was stirred for 2 min. Methoxylation was carried out about 30 min at 70 °C. Next, 40 μL N-(tert-butyl dimethylsilyl)-N methyltrifluoroacetamide and 1% tert-butyl dimethyl chlorosilane were mixed and derivatized at 70 °C for 1 h. Finally, the samples were analyzed by gas chromatography (Agilent7890B) and mass spectrometry (Agilent5977A) using HP-5MS column (30 m × 0.25 mm i.d. coated with 0.25 μm film thickness, Agilent). The GC temperature program was as follows: 50 °C for 1 min, heat up to 200 °C by 10 °C/min, 200 °C for 5 min, heat up to 220 °C by 5 °C/min, 220 °C for 10 min, heat up to 250 °C by 15 °C/min and maintain 10 min. The inlet temperature was 250 °C and the mass range was 35–400 MHz. The ion source chamber temperature was 230 °C, the transmission line temperature was 250 °C, and the electron energy is 70 eV.

### Fecal DNA extraction, shotgun metagenomic sequencing and data quality control

The QIAamp^®^ DNA Stool Mini Kit (Qiagen, Hilden, Germany) was used for DNA extraction from the fecal samples. The quality of the extracted DNA was assessed by 0.8% agarose gel electrophoresis, and the OD 260/280 was measured by spectrophotometry. All of the DNA samples were subjected to shotgun metagenomic sequencing by using a HiSeq 2500 instrument (Illumina, CA, USA) in the Novogene Company (Beijing, China). Libraries were prepared with a fragment length of ~300 bp. Paired-end reads were generated using 100 bp in the forward and reverse directions. The quality of the reads were controlled by FastQC and were subsequently aligned to the human genome to remove the host DNA fragments. The details of sequencing statistics were exhibited in Table S2, the values of reads number were quality controlled.

### Identification of microbial species, functional genes, and metabolic pathways

The shotgun reads were assembled into contigs and scaffolds using MEGAHIT (v1.0) [33] with the default parameters. For metagenomic species annotation (Table S3 and Table S4), the Bracken software was applied [34]. For metagenomic functional features and metabolic pathway annotation (Table S5), HUMAnN (v2.0) [15] was performed by using the UniRef90 database. More information was listed in “code availability”. Accordingly, we got the relative abundance of intestinal microbial taxonomic, gene families and metabolic pathway profiles. Differential abundance analysis was performed using Songbird [35], a compositionality-aware statistical method.

### Construction of metagenome-assembled genomes (MAGs) and reconstruction of a phylogenomic tree of MAGs

For metagenomic species analysis, MetaBAT (v1.0) [36] was applied to generate MAGs by binning shotgun reads (Table S6). After reassembling, each MAG was assigned to a reference genome if more than 80% of the sub-gene identified by Prodigal matched the same genome using BLASTn at a threshold of 95% identity over 90% of the gene length. If >80% of the genes from a MAG had the same taxonomic level of assignment, that MAG was identified as the same microbe. Recovered MAGs were subjected to phylogenomic reconstruction using PhyloPhlAn2 [37] under a high diversity setting (the “Prokaryotes Tree of life reconstruction” protocol). The resulting phylogenetic tree was visualized using iTOL (v4) [38].

### Integrated analysis of clinical and microbiome-derived features

The Mantel test [39] was performed to quantify the correlation between distance matrices of each pair of data types across subjects. The Adonis test [40] was performed to quantify the contribution of the subjects’ physical variables to the microbiome.

### Intestinal metabolites prediction and the GD characterized intestinal microbiome network construction

MelonnPan (Model-based Genomically Informed High-dimensional Predictor of Microbial Community Metabolic Profiles) pipeline [14] was used to predict the metabolite composition (Table S7) from microbiome sequencing data. At last, we constructed the network including the MAGs, metabolites and clinical indexes above by calculating the Spearman’s rank correlation coefficient among them and visualized the network by Cytoscape (v3.7.1) [41] software to exhibit the correlation between the intestinal microbiome and GD.

### Evolutionary analysis based on shotgun metagenomic data of gut microbiota

We employed the MIDAS (v1.0 Metagenomic Intra-Species Diversity Analysis System) to perform intestinal microbiota mutations annotation [42]. Briefly, a reference genome database including 33 species with the abundance more than 0.1% was constructed. Then the shotgun metagenomic sequencing reads were mapped to the database for intestinal species SNP calling. Then, the samples in the control group were set as the standard for bacterial mutation judgment of other samples in the GD groups to identify the significant difference SNPs among the groups (Table S8). The SNPs profiles (Table S9) for these intestinal microbes were deposited in GitHub: https://github.com/zhjch321123/Graves_Disease_Microbiome.git.

### The machine learning approach for disease-state classification and identification of potential GD-related biomarkers

Our machine learning analysis systematically exploited a total of four types of microbiome quantitative profiles: reference-based taxonomic species-level relative abundances by Bracken, reference-free taxonomic relative abundances of MAG and gene-family and pathway-relative abundances, and the presence and absence patterns of SNPs.

The random forest algorithm [43] was employed to train sample classifiers for discriminating disease states using a combined feature set with four types of microbiome profiles. We applied the R package “ranger” (v0.12.1) to implement the random forest algorithm in each classification task with the default hyperparameters except the number of trees was set as 5000. The prediction performance of RF models was evaluated with a fivefold cross-validation approach. We further validated the performance using the 50–50% training and testing splits. The final performance (accuracy) was compared across different cross-validation approaches and reported the accuracy in the 50% holdout test set in the results.

To determine how many features can maximize the model performance, we built disease classifiers using a series of reduced sets (e.g., *n* = 2, 4, 8, 16, 32, 64, 128, 256, etc.) of microbial features and compared their performance. The rationale is to observe the increase or peak in prediction accuracy when additional features are added into a classification model.

### Publicly available human gut metagenomes of seven metabolic diseases and the specificity of the GD biomarkers

To extend the significance of the present research, we performed a multi-cohort analysis across eight metabolic diseases, including: ankylosing spondylitis (SRP100575), liver cirrhosis (SRP011011 and ERP005860), colorectal cancer (ERP008729), PD (two cohorts including a European cohort (PDA) and a Chinese cohort (PDB), PRJNA433459 and ERP019674), rheumatoid arthritis (ERP006678) and type 2 diabetes (SRP008047). Meanwhile, we detected the specificity of the present GD biomarkers by performing the same annotation pipeline of the present research.

## Supplementary information

Supplementary Figures

Dataset 1

## Data Availability

The authors declare that the data supporting the findings of this study are available within the paper and its additional files. The sequence data reported in this paper have been deposited in the NCBI database (resequencing and metagenomic sequencing data: PRJNA602729, PRJNA602731, PRJNA602732, PRJNA638403, PRJNA638404 and PRJNA638405). More details about the figure construction and repeatable can be found in the document “Project analysis code and figure reproducibility” deposited in GitHub: https://github.com/zhjch321123/Graves_Disease_Microbiome.git.

## Code availability

All statistical analyses were performed using the R software. PCoA analysis was performed in R using the “ade4” package (v1.7.13). Considering the compositional nature of taxonomic and functional profiles of gut microbiome, Central log-ratio (CLR) transformation was performed prior to differential abundance analysis by the “zCompositions” package (v1.3.2). To perform CLR-transformation of raw relative abundances of taxa and functional genes, zero values in the raw matrix were replaced with the 1/10 of the minimum non-zero value in the matrix. Wilcoxon rank-sum test was employed to identify the differentially abundant microbial features (i.e., species-level, functional genes, and predicted metabolites) between each pair of healthy, GD I and GD II groups. The statistical significance was declared at a 0.05 nominal level of corrected *p* values with Benjamini–Hochberg (BH) method (for controlling the false discovery rate). Heatmaps were constructed using the “pheatmap” package (v1.0.12). For boxplot construction, the package “ggpubr” (v0.2.3) was used. The edges of the network were calculated by the Spearman’s rank correlation coefficients and visualized in Cytoscape v3.7.1 [41].
