# Correction to: Compositional and genetic alterations in Graves’ disease gut microbiome reveal specific diagnostic biomarkers

**DOI:** 10.1038/s41396-021-01040-7

**Published:** 2022-01-18

**Authors:** Qiyun Zhu, Qiangchuan Hou, Shi Huang, Qianying Ou, Dongxue Huo, Yoshiki Vázquez-Baeza, Chaoping Cen, Victor Cantu, Mehrbod Estaki, Haibo Chang, Pedro Belda-Ferre, Ho-Cheol Kim, Kaining Chen, Rob Knight, Jiachao Zhang

**Affiliations:** 1 Department of Pediatrics, University of California San Diego, La Jolla, CA USA; 2 Center for Microbiome Innovation, Jacobs School of Engineering, University of California San Diego, La Jolla, CA USA; 3 School of Food Science and Engineering, Hainan University, Haikou, China; 4 Department of Endocrinology, Hainan General Hospital, Hainan Affiliated Hospital of Hainan Medical University, Haikou, China; 5 Hubei Provincial Engineering and Technology Research Center for Food Ingredients, Hubei University of Arts and Science, Xiangyang, Hubei province China; 6 Key Laboratory of Food Nutrition and Functional Food of Hainan Province, Haikou, China; 7 Department of Bioengineering, University of California San Diego, La Jolla, CA USA; 8 Scalable Knowledge Intelligence, IBM Research-Almaden, San Jose, CA USA; 9 Department of Computer Science and Engineering, University of California San Diego, La Jolla, CA USA

**Keywords:** Microbiome, Biomarkers, Population genetics

Correction to: *The ISME Journal* 10.1038/s41396-021-01016-7, published online 2 June 2021

The original version of this article unfortunately contained a mistake in the affiliation of the first author.

Qiyun Zhu affiliations are:

^2^Department of Pediatrics, University of California San Diego, La Jolla, CA, USA

^3^Center for Microbiome Innovation, Jacobs School of Engineering, University of California San Diego, La Jolla, CA, USA

The original article has been corrected

