# Supplementary material for: Compositional and genetic alterations in Graves’ disease gut microbiome reveal specific diagnostic biomarkers
Source: ISME J. 2021 Jun 2;15(11):3399–411. doi: 10.1038/s41396-021-01016-7 (PMC8528855; doi:10.1038/s41396-021-01016-7)
Supplement: Supplementary file 1 — Supplementary Figures [file 41396_2021_1016_MOESM1_ESM.pdf]

Supplementary figures

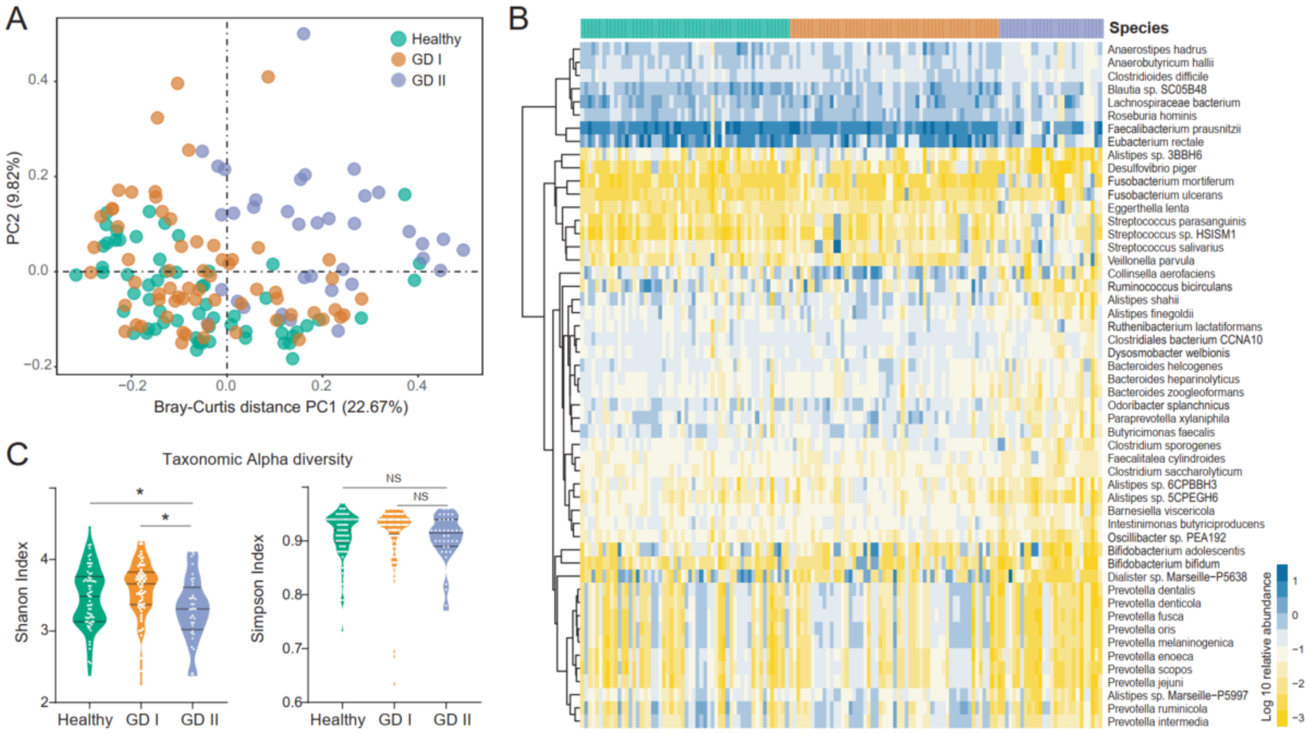

Figure S1. Comparative analysis of gut microbiome composition and diversity among the three groups: healthy controls (Health), mild Graves' patients (GD I) and severe Graves' patients (GD II): **A.** Principal coordinate analysis (PCoA) of Bray-Curtis distances among MAG profiles. Each dot represents a human subject and is colored by the host groups. **B.** Heatmap showing log10-transformed relative abundance of species-level markers associated with GD states (i.e. Healthy, GD I and GD II). **C.** Distribution of Shannon diversity indices of the three groups. Asterisks: statistical significance (* $p \leq 0.05$, NS, not significant)

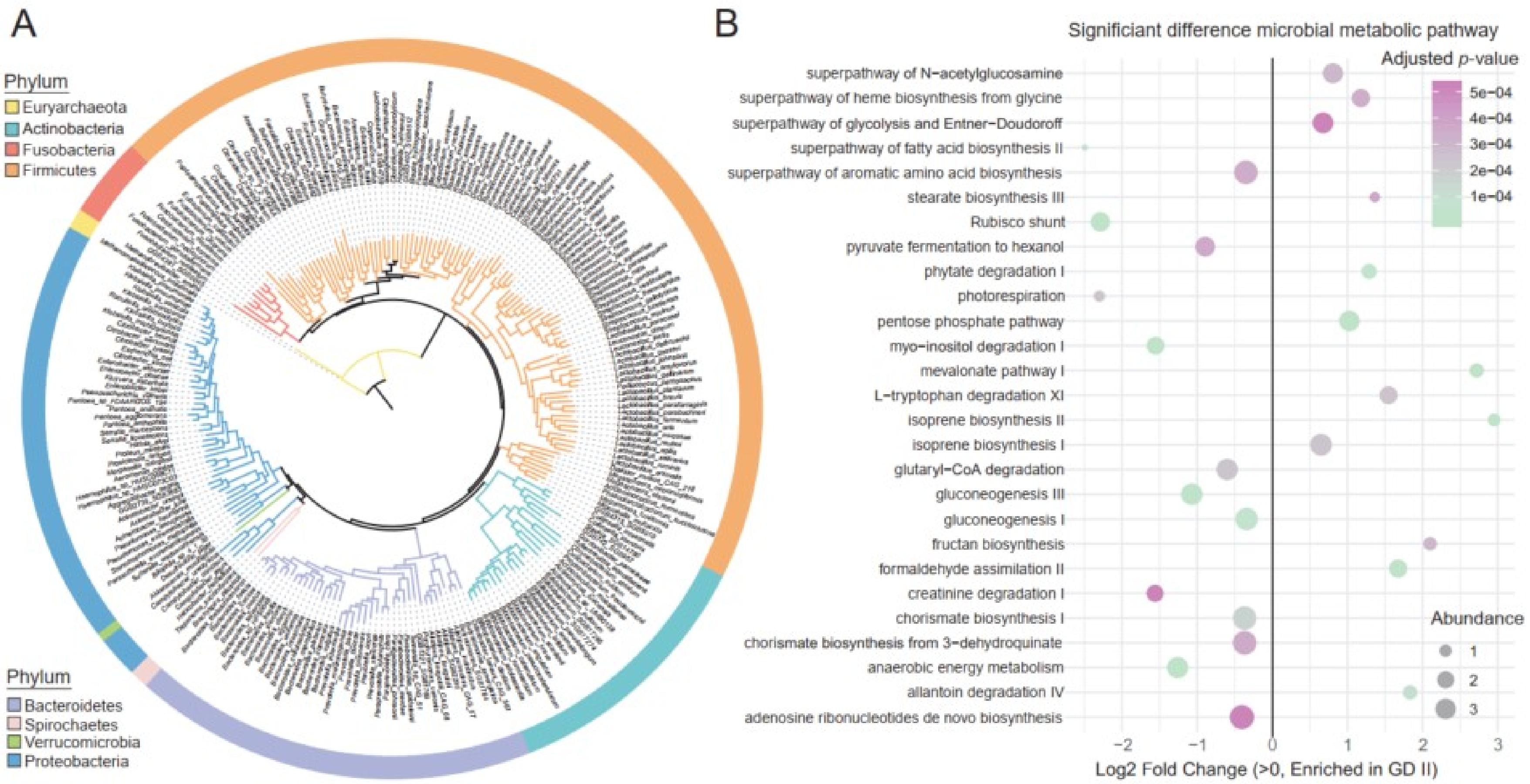

Figure S2. A. Phylogenomic tree of MAGs recovered from this study, with clades colored by phylum. **B.** The differentially enriched microbial metabolic pathways between the Healthy and GD II groups.

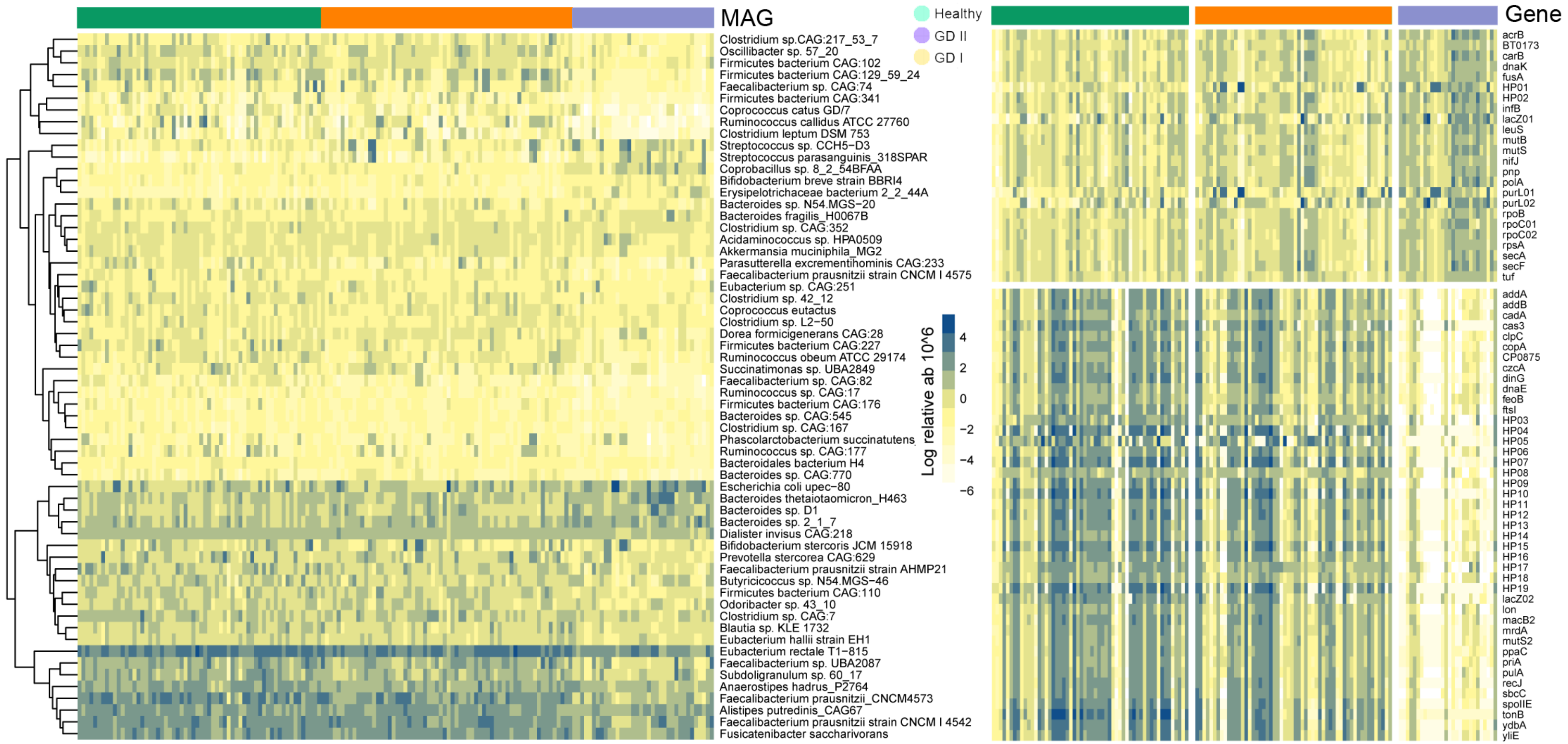

Figure S3. The differentially abundant MAGs and MAG-annotated genes in the gut microbiome among human host groups.

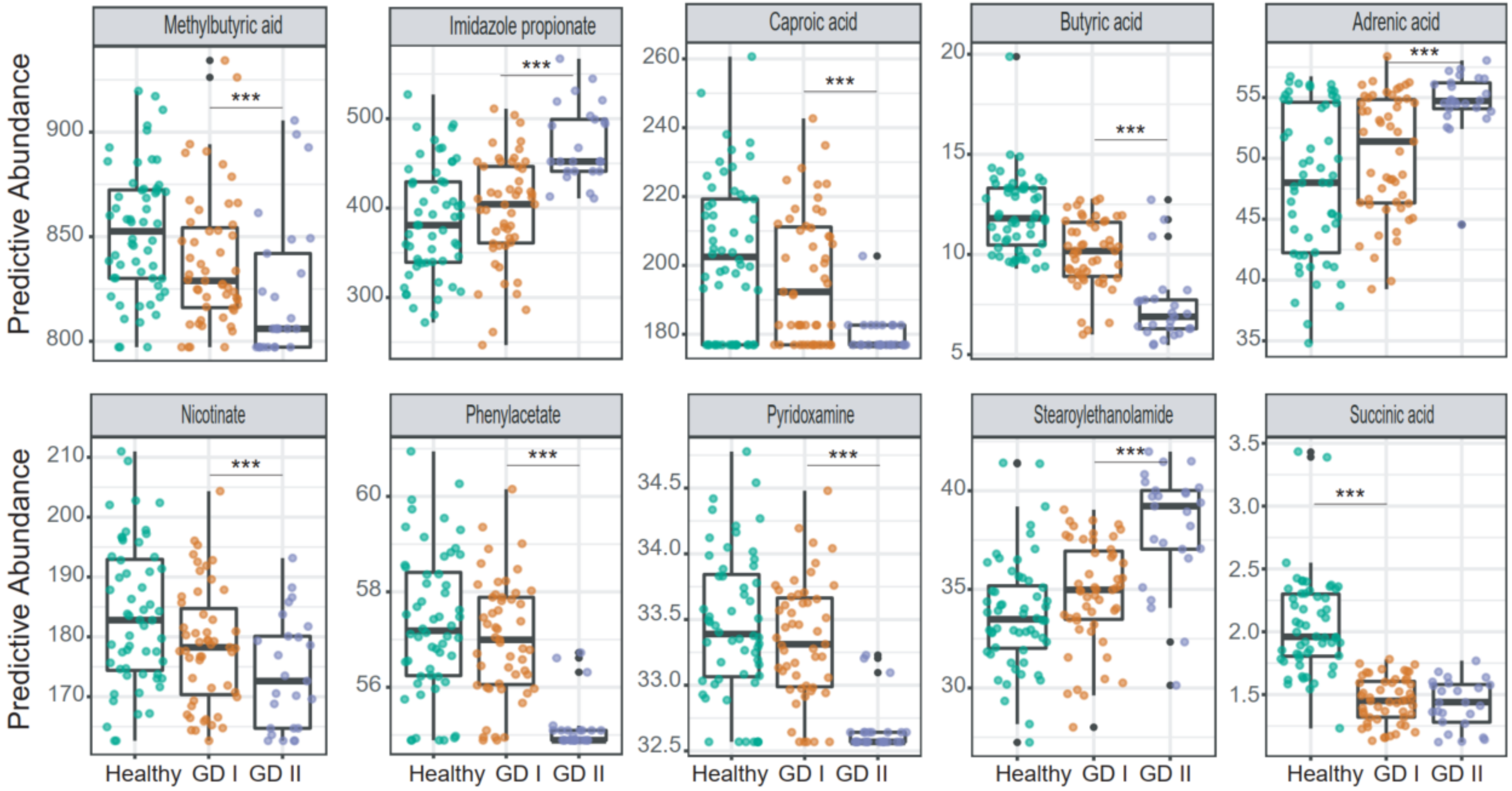

Figure S4. The differentially abundant metabolites in the gut microbiome between the healthy controls and severe Graves' patients (GD II). Asterisks: statistical significance (* $p \leq 0.05$, ** $p \leq 0.01$, *** $p \leq 0.001$).

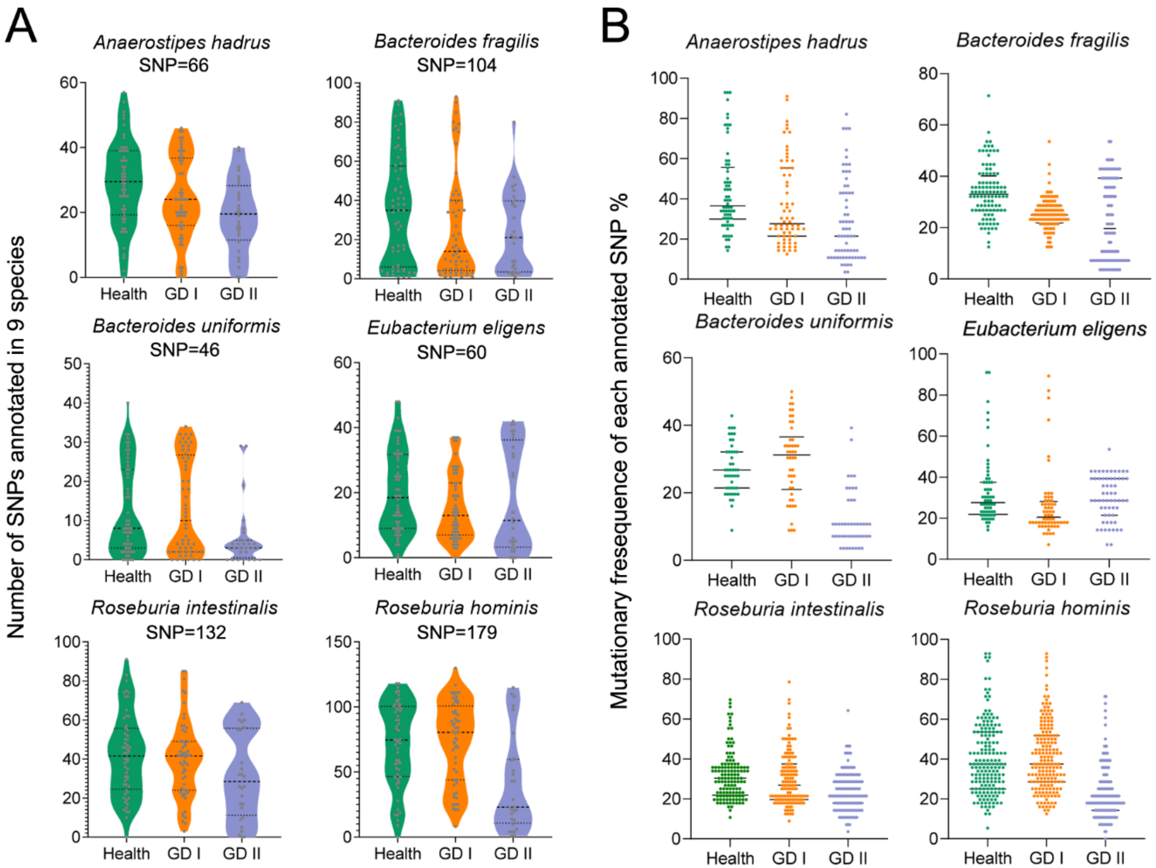

Figure S5. The SNP profiles of target species in each group. A. The violin plot shows the distribution of SNPs in a given microbial genome between hosts under different disease states. The number of single-nucleotide mutations (SNPs) in the six prevalent intestinal species ranged from 46 to 179. **B.** The mutational frequency of each SNPs annotated in the intestinal species.

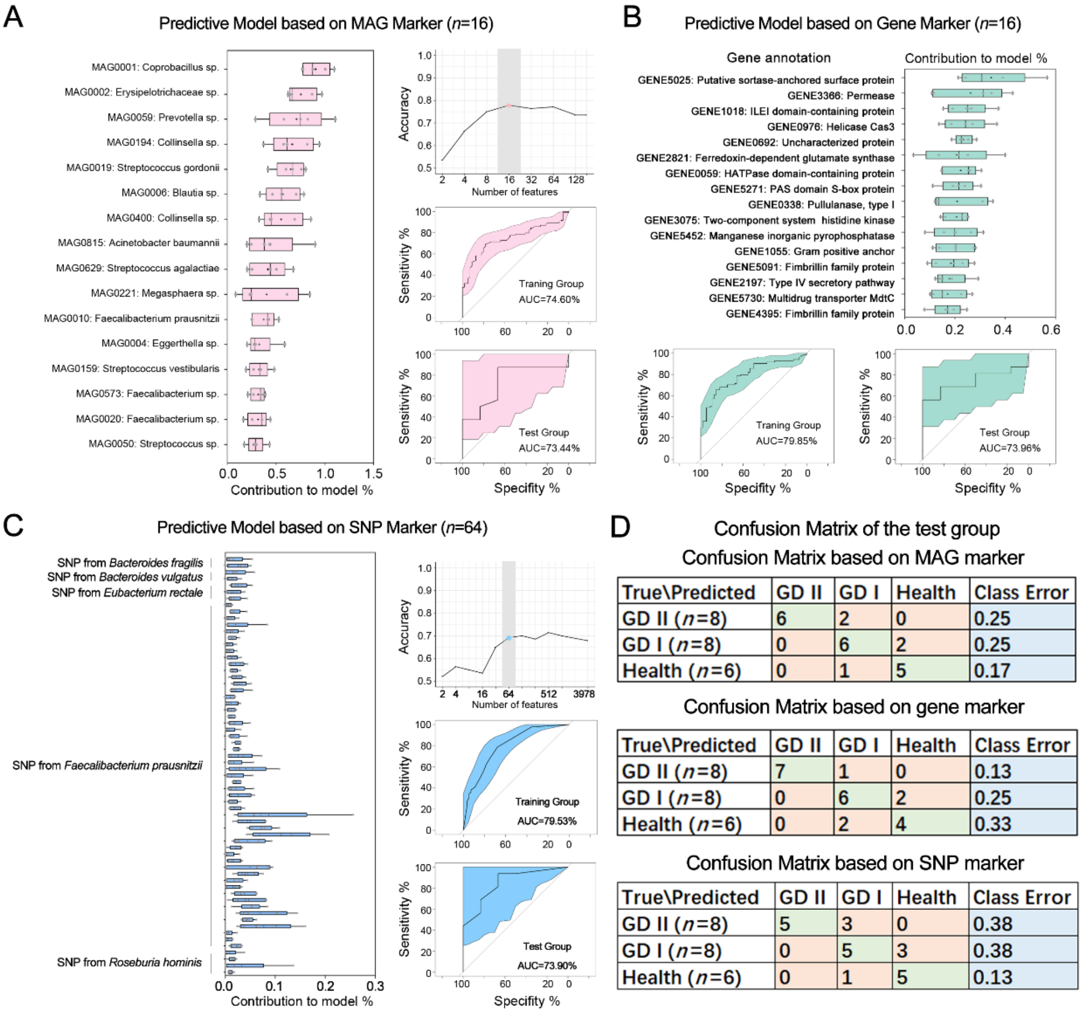

Confusion Matrix based on MAG marker

True\Predicted	GD II	GD I	Health	Class Error
GD II (*n*=8)	6	2	0	0.25
GD I (*n*=8)	0	6	2	0.25
Health (*n*=6)	0	1	5	0.17

Confusion Matrix based on gene marker

True\Predicted	GD II	GD I	Health	Class Error
GD II (*n*=8)	7	1	0	0.13
GD I (*n*=8)	0	6	2	0.25
Health (*n*=6)	0	2	4	0.33

Confusion Matrix based on SNP marker

True\Predicted	GD II	GD I	Health	Class Error
GD II (*n*=8)	5	3	0	0.38
GD I (*n*=8)	0	5	3	0.38
Health (*n*=6)	0	1	5	0.13

Figure S6. Identification of GD related intestinal microbial markers and its verification in the test group. We built predictive models based on the MAGs **(A)**, MAG-derived genes **(B)** and SNPs of the specific species markers **(C)**. The importance of the biomarkers was ranked according to their quantitative contribution to the predictive model. We also examined how many important features in a reduced model can maximize the prediction accuracy. The prediction performance both in the training and test splits was evaluated by the area under the receiver operating characteristic (AUC). **D.** The confusion matrix of the test split for further validation of the predictive accuracy of the important biomarkers derived from taxonomic profiles, MAG profiles or SNP profiles.

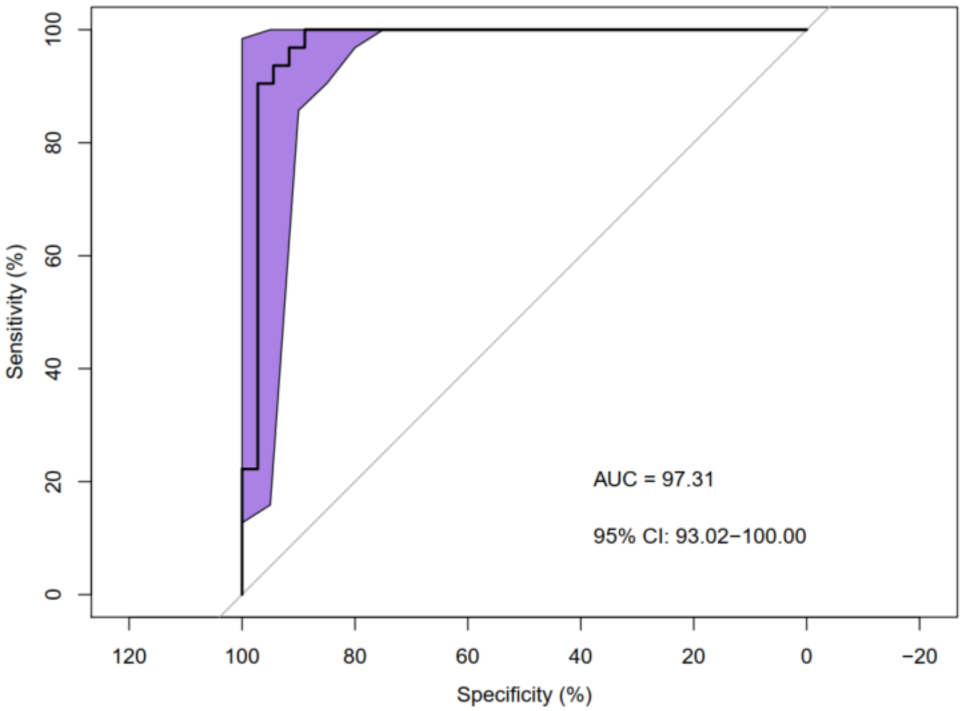

Figure S7. The ROC curve of the predictive model based on the five GD-specific biomarkers in the combined cohort of GD and PD patients. The AUC of the five markers reached 97.31% in the cohort.
